# Supplementary material for: Virtual screening of antimicrobial plant extracts by machine-learning classification of chemical compounds in semantic space
Source: PLoS One. 2023 May 15;18(5):e0285716. doi: 10.1371/journal.pone.0285716 (PMC10184910; doi:10.1371/journal.pone.0285716)
Supplement: Attachment — Submitted filename: RebuttalLetter.docx [file pone.0285716.s005.docx]

[PONE-D-23-09446]

Journal Requirements:

1. Please ensure that your manuscript meets PLOS ONE's style requirements, including those for file naming. The PLOS ONE style templates can be found at

https://journals.plos.org/plosone/s/file?id=wjVg/PLOSOne_formatting_sample_main_body.pdf and

https://journals.plos.org/plosone/s/file?id=ba62/PLOSOne_formatting_sample_title_authors_affiliations.pdf

**RESPONSE:** Thank you for your advice. We have checked the style requirements again, and uploaded figure files corrected by PACE.

2. Please note that PLOS ONE has specific guidelines on code sharing for submissions in which author-generated code underpins the findings in the manuscript. In these cases, all author-generated code must be made available without restrictions upon publication of the work. Please review our guidelines at https://journals.plos.org/plosone/s/materials-and-software-sharing#loc-sharing-code and ensure that your code is shared in a way that follows best practice and facilitates reproducibility and reuse.

**RESPONSE:** We have uploaded the code to GitHub, and added a description “R scripts and preprocessed literature data are available at https://github.com/yabuuchi-hiroaki/webvs” to “Data Availability” field.

3. Please remove your figures from within your manuscript file, leaving only the individual TIFF/EPS image files, uploaded separately. These will be automatically included in the reviewers’ PDF.

**RESPONSE:** We have removed the figures. We are sorry for forgetting to remove them.

4. Please review your reference list to ensure that it is complete and correct. If you have cited papers that have been retracted, please include the rationale for doing so in the manuscript text, or remove these references and replace them with relevant current references. Any changes to the reference list should be mentioned in the rebuttal letter that accompanies your revised manuscript. If you need to cite a retracted article, indicate the article’s retracted status in the References list and also include a citation and full reference for the retraction notice.

**RESPONSE:** Thank you for your advice. We have checked the reference list again.

Comments from Reviewer 1:

“Virtual screening of antimicrobial plant extracts by machine-learning classification of chemical compounds in semantic space” Author have applied semantic space and word embedding algorithm to develop machine learning for classification of antimicrobial compouds. This a new type methods to develop machine learning model for classification.

In my opinion - It is a well-written manuscript. Methods are written explaning every step. The results and figures are well explained. The discussion and conclusion explain very well the outcomes of this manuscript.

**RESPONSE:** Thank you for dedicating your time to review our manuscript and for the positive opinion.

Comments from Reviewer 2:

The manuscript entitled ‘Virtual screening of antimicrobial plant extracts by machine-learning classification of chemical compounds in semantic space’ described the evaluation of a new computational screening strategy for classifying bioactive compounds and plants in semantic space generated by word embedding algorithm.

The manuscript is well written. The study is relevant and important especially for researchers in countries where traditional knowledge about the medicinal uses of available plant has become limited or unavailable. In such situations applications like this can help explore the flora for drug discovery. It is also important for areas with rich traditional knowledge but where indigens are unwilling to give out information on important medicinal plants for research.

Unfortunately, the application does not address the issues of chemical and bioactive variation in plants due to factors such as geographical location of plant material, season of harvesting etc. that may affect predictions by the application.

**RESPONSE:** Thank you for dedicating your time to review our manuscript and for constructive comments. You have raised an important point in the last paragraph. We have incorporated your comments by **p.18, lines 284-287**.

Lines 236-238: A stronger reason HTS are uncommon is the cost involved

in setting one up (acquisition, installation and maintenance). Revise.

**RESPONSE:** Thank you for your advice. We agree with you and have incorporated this suggestion (**p.15, line 238**).

Lines 248-249: why should plants that were previous investigated be subjects to WEBVS in its large-scale exploration? Is it necessary?

**RESPONSE:** We wrote "appeared in previous studies" to mean somewhat described in the literature data. However, as you pointed out, this phrase give an impression that the activity of plant is already investigated in previous studies. We have amended the manuscript to avoid reader's confusion (**p.16, line 250**).

Line 264: What else is there to consider about the medicinal potential of *C. sieboldii* when the authors had indicated that the plant is in short supply therefore its removal from the Japanese Pharmacopoeia? Revise.

**RESPONSE:** Thank you for the suggestive question. We have rewritten the history and background of *C. sieboldii* with some references (from **p.16 line 259** to **p.17 line 263**), and specified the need of further research including clinical studies (**p.17 lines 265-266**).
